# Effect of Eye Movement Desensitization and Reprocessing (EMDR) on Depression in Patients With Myocardial Infarction (MI)

**DOI:** 10.5539/gjhs.v7n6p258

**Published:** 2015-04-16

**Authors:** Mohammad Behnammoghadam, Ali Karam Alamdari, Aziz Behnammoghadam, Fatemeh Darban

**Affiliations:** 1 Department of Nursing, Yasuj University of Medical Sciences. Yasuj, Iran; 2 Social Determinants of Health Research Center, Yasuj University of Medical Sciences. Yasuj, Iran; 3 Department of Psychology, Islamic Azad University, Yasuj, Iran; 4 Department of Nursing, Iranshahr University of Medical Sciences. Iranshahr, Iran

**Keywords:** depression, myocardial infarction (MI), eye movement desensitization and reprocessing (EMDR)

## Abstract

**Background:**

Coronary heart disease is the most important cause of death and inability in all communities. Depressive symptoms are frequent among post-myocardial infarction (MI) patients and may cause negative effects on cardiac prognosis. This study was conducted to identify efficacy of EMDR on depression of patients with MI.

**Methods:**

This study is a clinical trial. Sixty patients with MI were selected by simple sampling, and were separated randomly into experimental and control groups. To collect data, demographic questionnaire and Beck Depression Questionnaire were used. In experimental group, EMDR therapy were performed in three sessions alternate days for 45–90 minutes, during four months after their MI. Depression level of patients was measured before, and a week after EMDR therapy. Data were analyzed using paired –t- test, t–test, and Chi-square.

**Results:**

The mean depression level in experimental group 27.26± 6.41 before intervention, and it was 11.76 ± 3.71 after intervention. Hence, it showed a statistically significant difference (*P*<0.001). The mean depression level in control group was 24.53 ± 5.81 before intervention, and it was 31.66± 6.09 after intervention, so it showed statistically significant difference (*P*<0.001). The comparison of mean depression level at post treatment, in both groups showed statistically significant difference (*P*<0.001).

**Conclusion:**

EMDR is an effective, useful, efficient, and non-invasive method for treatment and reducing depression in patients with MI.

## 1. Introduction

Coronary disease is the most important cause of death and inability in all communities. The American Heart Association (AHA) reported that 17.6 million people in the United States have coronary heart disease, which, including 8.5 million with MI, and 10.2 million with angina pectoris (Lloyd-Jones D, Adams & Brown, 2010).

By 2007, these diseases have been first cause of the mortality among people >35 years old in Iran (Hasanpour et al., 2007), and 40% total mortalities. As per the study conducted by Vyzshfar (2002), 120–140 thousands persons are dying annually for this reason. World Health Organization of the United Nations (WHO) notified the depression as 4^th^ generalized health problem (Modabernia et al., 2001). According to these studies, the depression following MI is an independent and potential serious reason of mortality, among the patients with MI. Incidence of the depression symptoms, following MI is a common psychological problem. Depressive symptoms are widespread among post-MI patients and may cause effects on cardiac prospects (Bagherian et al., 2011). In 40–60% patients, the manifest clinical symptoms of depression are seen. The gross risk (without controlling other agents of the risk) of the mortality, six-months after MI, in patients with depression is six times more than those without it. This high risk of the mortality is observed until 18 months after MI. It is evident that for preventing, or taking the subsequent therapeutic measures, it is important to diagnosis the depression in the patients with MI, especially identify the hospitalized patients with high-risk of incidence, the depression symptoms following MI.

Preventive implementations and therapeutic methods for post-MI depression can decrease risk of death and acceptance of therapeutic methods and rehabilitation, promote and maintain health level and quality of life, prevent development of risky behaviors, and finally it can prevent negative outcomes of depression patients with myocardial infarction

There are many methods for treating the depression following MI, such as, cardiac rehabilitation, social support, behavioral-cognitive therapy, and using anti-depression drugs (Bagherian et al., 2011). It has been shown that some anti- depression, anxiety drugs could result in the risk of the cardiac dysrhythmia and sudden death (Harry et al., 2003).

Considering results of the study by Behnammoghadam et al., (2013), it can be said that non-pharmacological methods, which are cheap and non-invasive, can be used for controlling and treating depression of patients with MI, and they do not have any side effects.

Behavioral-cognitive techniques, such as, EMDR are among the therapeutic methods used for treating the depression. This new method is safe and without negative side effects, and only using the rapid and regular eye movements of the patient (Behnammoghadamet al., 2015). Schneider et al., showed that after EMDR therapy, there was a significant recovery in hospital depression, anxiety, and stress symptoms, followed by post-traumatic stress disorder in a patient with co-morbid epilepsy (Schneider et al., 2005).

Tavanti et al., compared two therapeutic methods of Sertraline and EMDR in treating the symptoms of depression and anxiety. Their results showed that both Sertraline and EMDR were significant reductions in PTSD symptoms (*P*<0.001), and EMDR would obtain faster recovery than Sertraline (Tavanti et al., 2008). Arabia et al., conducted a study on treatment of Post-Traumatic Stress Disorder (PTSD) and anxiety and depression symptoms in survivors of life-threatening cardiac events. The findings of their study indicated the mean depression score of 17.14 before intervention in these patients. After employing EMDR procedure, the score decreased to 6.38, which indicate statistically significant difference (Arabia et al., 2011).

## 2. Materials and Methods

This study was a clinical trial conducted to identify efficacy of EMDR on the depression of patients with MI. Firstly, the patients were assessed according to Beck Depression Questionnaire and included in the study, if they were depressive (score >17). They divided into two groups randomly, as experimental and control group, each with 30 subjects.

For experimental group, depression was measured before and after using the therapeutic method by Beck Depression Questionnaire (pre- and post-test). This therapeutic method was performed at three sessions per 45–90 min for one week to experimental group during four months, after their MI in Heart and Vessel Clinic, and after one week, their depression score studied again.

For control group, at first session, demographic characteristics and Beck Depression Questionnaire completed; then after four months and at second session, Beck Depression Questionnaire completed again.

MI diagnosis by a cardiologist, stability of the hemodynamic condition of the patient, literacy, age group of 30–70 years, lack of psychological diseases, addiction, strabismus, and visionary problems were the criteria to consider the study, while inability to tolerate EMDR procedure, and lack of cooperation with therapist were the exclusion criteria. For data collection, two instruments, including demographic questionnaire, and Beck Depression Inventory, were used. The demographic characteristics were age, gender, marital status, education level, and smoking experience. To measure depression level, BDI instrument was applied. BDI contains 21 questions. Scoring system of the responses was as “not at all” (0), “minimum” (1), “moderate” (2), and “severely” (3). The BDI has a maximum score of 63 where: 0–9, 10–16, 17– 29, and >30 indicate, not at all, minimum, moderate, and severe levels of depression, respectively.

### 2.1 Protocol of EMDR Therapy

The EMDR therapy included eight phases. Each session time was 45–90 minutes. In the reprocessing phases, the patient was instructed to recognize a picture that represents the most terrible part of the cardiac incident; a negative unreasonable self-belief related with the picture; a positive adaptive cognition, emotions, and employee body sensations. Then, at first, focusing on the picture, negative belief, and sensation, the patient was guided, according to standardized protocols to simultaneously move his eyes back and forward, following the therapist’s finger as they moved across their field of vision for a set of about 24–36 seconds. After the set, the patient reported any new associations that may have emerged. Such associations, generally, became the focus of the next set of double attention, or were guided by the physician. This procedure continued until the target memory was desensitized. After that, more eye movement sets were used, while the patient was thinking of a recognized adaptive belief. This was repeated until the new statement felt proper to the patient, and until all physical disorder was dissipated. Over the sessions, this treatment method was used to address memories of the cardiac incident, and associated present triggers, as well as anticipatory anxiety related to possible future incidents (Shapiro, 2001).

The data collected in experimental and control group were analyzed using the SPSS-16 software, through the descriptive and inferential statistic tests, such as, independent *t* (for comparing mean depression level in two groups), paired *t*-test (for comparing the mean depression level before and after intervention), and Chi-square test (for investigating demographic characteristics of the samples).

The author started data collecting process after obtaining permission from Ethics Committee of Yasuj University of Medical Sciences (registration No. 28.20.6190), and registering it in Iran Clinical Trial Center (registration No. of IRCT2012090610752N1); received written informed consent form from all subjects; and received official permission from the concerned authorities. There was no obligation for the subjects to participate in this work and they were ensured about confidentiality of all information.

## 3. Results

The results of this study showed that the two groups indicate no significant statistical difference, and were homogenous in terms of demographic properties, such as, gender, education level, marital status, except two groups, which had significant difference in terms of smoking (*P* < 0.001). The mean age of the samples was 50.97 ± 8.25 and all of them were placed in age range of 35–70.

**Table 1:** Comparison of distribution of qualitative demographic variable in experimental and control groups

		Experimental N(%)	Control N(%)	P-value
Gender	Male	25(83.3)	25(83.3)	0.635
	Female	5(16.7)	5(16.7)	0.931
Level of education	High school	15(50)	15(50)	
	Bachelor degree	9(30)	10(33.3)	
	Higher than Bachelor degree	6(20)	5(16.7)	
Marital status	Single	2(6.7)	3(10)	0.5
	Married	28(93.8)	27(90)	
Smoking	Yes	20(66.7)	7(23.3)	0.001
	No	10(33.3)	23(76.7)	

**Table 2:** Comparison of the mean scores of depression in experimental and control groups

	Experimental Mean(SD)	Control Mean(SD)	P-value
Pre treatment	27.26(6.41)	24.53(5.81)	0. 001
Post treatment	11.76(3.71)	31.66(6.09)	0. 001
Note: Significant at α=0.05.			

Mean depression levels in experimental group were 27.26 ± 6.41 and 11.76 ± 3.71 before and after intervention; so it showed a statistically significant difference (*P*<0.001). Mean depression levels in control group were 24.53 ± 5.81 and 31.66 ± 6.09 before and after intervention; so it showed a statistically significant difference (*P*<0.001). The comparison of mean depression level at post treatment, in both groups showed a statistically significant difference (*P*<0.001). 

## 4. Discussion

The results of this study showed that there was a significant reduction in the depression symptoms of mean score Beck scale post-test to value the depression, in experimental group compared to its pre-test and in control group (*P*<0.001); and after ending the therapeutic sessions, patients in experimental group experienced much less depression so that they were at normal range of depression.

By comparing the mean depression level before and after participating in both the groups, effectiveness of EMDR in reducing depression is known. So, the findings confirm that EMDR results in reducing the depression symptoms of the patients with MI. Schneider et al., showed that EMDR yielded a significant recovery in the hospital depression, and anxiety and PTSD symptom with post-traumatic stress disorder in a patient with co-morbid epilepsy. Their results were consistent with present study and confirm the same.

Arabia et al., conducted a study on treatment of PTSD and anxiety and depression symptoms in survivors of life-threatening cardiac events. The findings of their work indicate the mean depression score of 17.14, before intervention in these patients. After employing EMDR procedure, the score decreased to 6.38, which indicates statistically significant difference (*P*< 0.001). These results, correspond with other randomized-controlled studies, found that EMDR treatment had a concurrent effect in reducing depressive symptoms in survivors of traumatic events, who developed PTSD symptoms (van der Kolk et al., 2007).

Abbasnejad et al., conducted a work on efficiency of EMDR in reducing unpleasant feelings resulting from Bam earthquake, and found that the mean depression score before the intervention in experimental group and after the intervention were 33.51 and 16.42, which indicates statistically significant difference (*P* < 0.001) (Abbas Nejad et al., 2006).

Narimani et al. (2009) compared the effect of EMDR and behavioral-cognitive method on treating PTSD symptoms in the veterans. Their findings showed that both the methods result in a significant reduction in amount of the hospital depression, but they had no significant difference in terms of the amount of reducing the depression. These findings are consistent with the results of Narimani and Rajabi (2009).

Power et al. (2002) found that both the EMDR and exposure therapy plus cognitive restructuring produced significant improvement; EMDR was more beneficial for depression, and required fewer treatment sessions.

One of the main limitations of the present study was little number of female subjects.

Finally, nurses can use this new and effective method for treatment of depression. EMDR, a standard protective program, is used in clinics and hospitals to treat patients in depression.

It is recommend that the results were used for clinical practice and further studies with larger samples can be taken up on other cases of depression.

## 5. Conclusions

The results showed that EMDR is effective in reducing and treating the depression of the patients with MI. The nurses at critical care units can use EMDR as a standard, new, and economical method for treating, or reducing the depression in patients with MI.
